# What is the added value of specialist radiology review of multidisciplinary team meeting cases in a tertiary care center?

**DOI:** 10.1007/s00330-024-10680-0

**Published:** 2024-03-15

**Authors:** Ömer Kasalak, Jeroen Vister, Marcel Zorgdrager, Reina W. Kloet, Jan P. Pennings, Derya Yakar, Thomas C. Kwee

**Affiliations:** Department of Radiology, Nuclear Medicine and Molecular Imaging, University Medical Center Groningen, University of Groningen, Hanzeplein 1, P.O. Box 30.001, 9700 RB Groningen, The Netherlands

**Keywords:** Clinical conference, Interdisciplinary communication, Workload

## Abstract

**Purpose:**

Multidisciplinary team meetings (MDTMs) are an important component of the workload of radiologists. This study investigated how often subspecialized radiologists change patient management in MDTMs at a tertiary care institution.

**Materials and methods:**

Over 2 years, six subspecialty radiologists documented their contributions to MDTMs at a tertiary care center. Both in-house and external imaging examinations were discussed at the MDTMs. All imaging examinations (whether primary or second opinion) were interpreted and reported by subspecialty radiologist prior to the MDTMs. The management change ratio (MC_ratio_) of the radiologist was defined as the number of cases in which the radiologist’s input in the MDTM changed patient management beyond the information that was already provided by the in-house (primary or second opinion) radiology report, as a proportion of the total number of cases whose imaging examinations were prepared for demonstration in the MDTM.

**Results:**

Sixty-eight MDTMs were included. The time required for preparing and attending all MDTMs (excluding imaging examinations that had not been reported yet) was 11,000 min, with a median of 172 min (IQR 113–200 min) per MDTM, and a median of 9 min (IQR 8–13 min) per patient. The radiologists’ input changed patient management in 113 out of 1138 cases, corresponding to an MC_ratio_ of 8.4%. The median MC_ratio_ per MDTM was 6% (IQR 0–17%).

**Conclusion:**

Radiologists’ time investment in MDTMs is considerable relative to the small proportion of cases in which they influence patient management in the MDTM. The use of radiologists for MDTMs should therefore be improved.

**Clinical relevance statement:**

The use of radiologists for MDTMs (multidisciplinary team meetings) should be improved, because their time investment in MDTMs is considerable relative to the small proportion of cases in which they influence patient management in the MDTM.

**Key Points:**

• *Multidisciplinary team meetings (MDTMs) are an important component of the workload of radiologists.*

• *In a tertiary care center in which all imaging examinations have already been interpreted and reported by subspecialized radiologists before the MDTM takes place, the median time investment of a radiologist for preparing and demonstrating one MDTM patient is 9 min.*

• *In this setting, the radiologist changes patient management in only a minority of cases in the MDTM.*

## Introduction

The workload of radiologists has considerably risen over the past decades (both in terms of volume, complexity, and shortened turnaround times) and is expected to keep on increasing for the foreseeable future [1, 2]. Work overload may lead to burnout in radiologists and has been reported to be associated with diagnostic errors [3–8]. Therefore, it is desirable to maintain an adequate balance between workload and radiologist staffing.

Considering the increasing workload and the strive to deliver value-based healthcare (i.e., improving patient-relevant outcomes without increasing costs [9]), radiologists may have to reassess which of their activities truly contribute to patient management and outcomes, and which ones are less likely to do so. Radiologists take on multiple tasks in clinical practice [10], including participation in multidisciplinary team meetings (MDTMs).

The number of MDTMs has increased considerably over the past few years, and they contribute significantly to the overall workload [11–13]. MDTMs are generally regarded as beneficial to patient care [12–14]. However, there is a lack of studies that have actually investigated to what extent the participation of radiologists in MDTMs benefits patients. It remains unknown how often the participation of subspecialized radiologists in MDTMs changes patient management, particularly in hospitals in which all imaging studies are already routinely interpreted and reported by subspecialized radiologists before the MDTM takes place. This knowledge may be helpful in improving the efficiency of MDTMs from the perspective of the radiologist.

The purpose of this study was therefore to investigate how often subspecialized radiologists change patient management in MDTMs at a tertiary care institution.

## Materials and methods

### Study design

This study was approved by the institutional review board. The requirement for informed consent was waived, as no individual patient data were used. Six radiologists (one chest radiologist (Ö.K.), two abdominal radiologists (M.Z., D.Y.), two neuroradiologists (J.V. and R.W.K.), and one musculoskeletal radiologist (T.C.K.)) recorded their time investment and contribution to MDTMs in which they participated at a tertiary care center between November 2021 and October 2023.

### MDTMs

Patients whose imaging examinations have to be demonstrated by a radiologist in an MDTM in our hospital are put on a digital list by their treating physician. This digital list is available to the radiologist at least 1 day prior to the MDTM. It occasionally contains specific questions that may be of concern to the radiologist (e.g., “Is this pulmonary nodule suspicious for malignancy or not?”), but this is not standardized. The radiologist is (implicitly) supposed to demonstrate the relevant imaging examinations, to engage in clinical reasoning and decision-making when appropriate, and to answer any questions from clinicians during the MDTM. All radiologists in our hospital have one or more subspecialties and participate in MDTMs that match their subspecialty. Both in-house and external imaging examinations were discussed at the MDTMs. All imaging examinations (whether primary or second opinion) were interpreted and reported by a subspecialty radiologist prior to the MDTMs.

### Radiologists’ time investment, contribution, and influence on patient management

For each MDTM, the radiologist recorded the total number of cases whose imaging examinations were prepared for demonstration, the time needed to prepare the MDTM (excluding interruptions (such as phone calls) and excluding reporting time for imaging examinations that had not been reported yet by an in-house subspecialty radiologist), the time spent on attending the MDTM, the number of questions that were asked to the radiologist in the MDTM, and the number of cases in which the radiologist’s input in the MDTM changed patient management. A case was considered to have management change if either additional imaging, biopsy, treatment, or follow-up plans were changed because of the input of the radiologist in the MDTM, beyond the information that was already provided by the in-house (primary or second opinion) radiology report. Examples of management changes are performing follow-up CT after 3 months instead of performing CT-guided biopsy of a lung lesion, refraining from pancreaticoduodenectomy because of the detection of metastases, performing an additional MRI to assess the cervical spinal cord in a patient with metastatic disease in the cervical spine, and ultrasound-guided biopsy instead of short-term follow-up MRI of a soft tissue lesion to exclude malignancy. No differentiation was made between “major” and “minor” management changes.

### Data analysis

Results were descriptively analyzed. The management change ratio (MC_ratio_) of the radiologist was defined as the number of cases in which the radiologist’s input in the MDTM changed patient management as a proportion of the total number of cases whose imaging examinations were prepared for demonstration in the MDTM (i.e., percentage of studies where management was changed following primary or second opinion reading by a subspecialty radiologist). An MC_ratio_ of 0% indicates that the radiologist did not change patient management in any case, whereas an MC_ratio_ of 100% indicates that the radiologist changed patient management in every case in the MDTM. A Kruskal–Wallis test with post hoc Conover test was performed to assess for any differences in MC_ratios_ among the different types of MDTMs (provided the sample size was ≥ 5). *p*-values < 0.05 were considered statistically significant. Statistical analysis was done using MedCalc version 17.2 Software (MedCalc).

## Results

### MDTMs

The six radiologists recorded their time investment and contribution to a total of 68 MDTMs, namely 19 bone and soft tissue, 14 thoracic oncology, 10 head and neck oncology, 9 hepatobiliary, 4 gastrointestinal oncology, 3 gynecologic oncology, 3 thyroid, 3 urologic oncology, 2 adult neuro-oncology, and 1 neurodegenerative MDTM(s). A total of 1138 cases were prepared by the radiologists for these 68 MDTMs, with a median of 17 patients per MDTM (interquartile range [IQR] 13–21 patients). The imaging examinations of 112 (9.8%) of these 1138 cases were ultimately not discussed in the MDTMs.

### Radiologists’ time investment, contribution, and influence on patient management

The total time required for preparing and attending all 68 MDTMs was 11,000 min, with a median of 172 min (IQR 113–200 min) per MDTM, and a median of 9 min (IQR 8–13 min) per patient (Table 1). Overall, the preparation time for an MDTM was 1.4 times longer than the duration of the MDTM itself (6492 min vs. 4608 min, for all 68 MDTMs). A total of 191 questions were asked to the radiologists in the 68 MDTMs, with a median of 2 questions per MDTM (IQR 1–4 questions), and a median of 0.14 questions per patient (IQR 0.06–0.25 questions). The radiologists’ input changed patient management in 113 out of 1138 cases, corresponding to an MC_ratio_ of 8.4%. The median MC_ratio_ per MDTM was 6% (IQR 0–17%). The MC_ratios_ varied widely, both within the same type of MDTM and between different MDTMs (Table 2). The MC_ratios_ for head and neck oncology (median of 22.5%) and hepatobiliary (median of 14%) MDTMs were significantly higher (*p* < 0.05) than those for bone and soft tissue (median of 5%) and thoracic oncology (median of 0%) MDTMs (*p* < 0.05).

**Table 1:** Time spent on preparing and attending the MDTMs

	Preparation time (minutes)	MDTM time (minutes)	Total time (minutes)
All 68 MDTMs together	6492	4608	11,000
Per MDTM (median with IQR)	94 (61–126)	65 (43–90)	171 (113–200)
Per patient (median with IQR)	5 (4–7)	4 (3–5)	9 (8–13)

**Table 2:** MC_ratios_ for the different MDTMs. An MC_ratio_ of 0% indicates that the radiologist did not change patient management in any case, whereas an MC_ratio_ of 100% indicates that the radiologist changed patient management in all cases in the MDTM

MDTM	No	MC_ratio_	
		Median	Range
Bone and soft tissue	19	5%	0–19%
Thoracic oncology	14	0%	0–18%
Head and neck oncology	10	22.5%	11–30%
Hepatobiliary	9	14%	0–33%
Gastrointestinal oncology	4	5%	0–35%
Gynecologic oncology	3	0%	0–14%
Thyroid	3	27%	0–29%
Urologic oncology	3	0%	-
Adult neuro-oncology	2	0.5%	0–1%
Neurodegenerative	1	20%	-

## Discussion

The results of this study show that the participation of radiologists in MDTMs is time-consuming, given the fact that the median time required to prepare and attend an MDTM was 172 min (for a median of 17 patients per MDTM). Interestingly, preparing an MDTM generally took 1.4 times longer than the duration of the MDTM itself. Overall, the input of radiologists in MDTMs changed patient management in only a small minority (8.4%) of all cases compared to the information that was already available in the in-house (primary or second opinion) radiology report. If influencing patient management is regarded as the most important metric, the efficiency of MDTMs can be considered low from the perspective of the radiologist. Interestingly, the MC_ratios_ varied widely, both within the same type of MDTM and between different MDTMs, with significantly higher overall MC_ratios_ for some MDTMs. This may reflect observer variation between the different subspecialty radiologists (e.g., due to differences in accuracy and the use of different diagnostic thresholds, which may be influenced by several factors such as previous training and experience, and time pressures), and differences in the quality of the in-house radiology reports. The latter may also have been affected by the quality of the imaging requests of the referring physicians, but that topic was beyond the scope of the present study.

It should be noted that the participation of a radiologist in an MDTM may have more potential benefits than influencing patient management. A radiologist can be useful by merely demonstrating the medical images in the MDTM setting, and occasionally answering clinicians’ questions. Rereview of medical imaging for an MDTM may serve as a method of quality control (with opportunities for effective peer review, oral or written feedback to primary and secondary reporters, and clinical audit), provide reassurance (for which there is no management change) as to appropriate management particularly when the primary reporters were not members of the MDTM (considered to be a valuable part of clinical auditing and identifying good practices), provide educational opportunities (e.g., the radiologist may receive pathology feedback to medical imaging interpretations, and radiologists may educate clinical colleagues about the use of medical imaging), increase contact with clinical colleagues, improve the “visibility of the radiologist,” and inform the radiologists about new protocols and research developments. Although there are MDTM guidelines for radiologists [15], definite and quantifiable objectives for radiologists to achieve by participation in MDTMs have not yet been established by professional societies or guidelines. It also remains unclear whether it is efficient to achieve certain objectives by means of participation in an MDTM compared to other approaches. For example, it can be argued that it is far more efficient for the radiologist to obtain pathology feedback by looking up pathology reports in the electronic patient files than spending a median of 9 min per patient to be informed about the pathological diagnosis in an MDTM. MDTMs have no predefined learning objectives either, and it can be contended that other dedicated educational methods may be more efficient. Cost-effective improvement of patient outcomes may be regarded as the goal of an MDTM. However, to the best of our knowledge, there are no randomized controlled trials that have proven that the participation of radiologists in MDTMs improves patient outcomes.

On a personal note, the authors believe that participation of radiologists in MDTMs can absolutely be beneficial to patient care. However, radiologists face increasing workload demands [1, 2], and burnout is prevalent in the specialty, with self-reported burnout in approximately one out of three radiologists in recent survey studies [16, 17]. The participation of radiologists in MDTMs should therefore be optimized. For example, treating physicians may select only those cases for preparation by the radiologist in which the in-house radiology report is deemed incomplete or not sufficiently clear to make patient management decisions, and formulate specific questions beforehand to be answered by the radiologist in the MDTM. This may increase the value and efficiency of the radiologists’ MDTM participation. This will also increase the meaningfulness of the work for radiologists and potentially decrease burnout. It will also improve “positive visibility of the radiologist” because the radiologist’s input in the MDTM will become more clinically important, rather than just acting as a colleague demonstrating medical images. In previous work by Balasubramanian et al [18], several other potential ways to improve the efficiency of MDTMs were proposed (Table 3). Patients may also be put on the MDTM list of patients to be discussed with the radiologist for other reasons (e.g., quality control, education). However, we believe there should first be consensus about the exact objectives that have to be achieved by radiologists at MDTMs in relation to the growing number of other tasks that they must also fulfill (e.g., diagnostic image interpretation and interventional procedures), and which should be prioritized or not to keep health care delivery efficient and sustainable. Our results also provide an estimate of the time a radiologist requires per patient that is put on an MDTM list (median of 9 min per patient), which may be used by healthcare policymakers and for reimbursement purposes.

**Table 3:** Several potential ways to improve the efficiency of MDTMs, as described by Balasubramanian et al [18]

Only discuss complex cases in the MDTM
Handle standard cases through established protocols outside of the MDTM
A pre-MDTM could identify cases that could be managed via treatment algorithms/pathways without being discussed in the MDTM and remove cases where all the relevant information or investigations are incomplete
Improved integration of information technology (including artificial intelligence) in the MDTM workflow
Image interpretation and reporting by subspecialty radiologists using standard templates

Previous works have already shown that MDTMs require significant time investments from radiologists [11–13]. Numerous previous studies have shown that reinterpretation of imaging examinations by subspecialty radiologists may benefit diagnostic accuracy and may impact patient management compared to original interpretations by general radiologists [19–24]. However, in our hospital, the referring physician receives all in-house second opinion reports before the MDTM takes place. So far, there has been a lack of studies that have quantified the yield of the radiologist’s participation in an MDTM after this second opinion reading has already been done.

The present study had some limitations. First, it only evaluated the radiologist’s influence on patient management in MDTMs. Future work is required to quantify other potential benefits of MDTM participation (such as educational opportunities and “visibility of the radiologist”) in relation to the time invested. In addition, a randomized controlled trial should be performed to determine the effect of the participation of radiologists in MDTMs on patient outcome. Second, it remains unclear if the changes in patient management that were caused by the radiologists’ input in the MDTMs were justified. Third, the determination of patient management changes may have been subject to some degree of subjectivity. Fourth, the results of this study apply to a tertiary care center in which subspecialty radiologists participate in MDTMs. It remains unclear if the results also apply to MDTMs done in other types of hospitals or by general radiologists. Fifth, several other types of MDTMs (such as pediatric, breast, cardiac, and interventional radiology) were not included in this study. Sixth, the MC_ratio_ metric we introduced has not been previously described or validated. Seventh, this work should not be regarded as a clinical audit, but more as a service evaluation.

In conclusion, radiologists’ time investment in MDTMs is considerable relative to the small proportion of cases in which they influence patient management in the MDTM. The use of radiologists for MDTMs should therefore be improved. Future studies on this topic should also consider other factors such as uniformity of care according to evidence-based practice, validate the MC_ratio_ metric, and focus on patient outcome measures.

## Abbreviations

IQR: Interquartile range
MDTM: Multidisciplinary team meeting

## Declarations

### Guarantor

The scientific guarantor of this publication is Ömer Kasalak.

### Statistics and biometry

No complex statistical methods were necessary for this paper.

### Methodology

• prospective

• experimental

• performed at one institution
